# Nematode-free agricultural system of a fungus-growing termite

**DOI:** 10.1038/s41598-019-44993-8

**Published:** 2019-06-20

**Authors:** Natsumi Kanzaki, Wei-Ren Liang, Chun-I Chiu, Ching-Ting Yang, Yen-Ping Hsueh, Hou-Feng Li

**Affiliations:** 1 Kansai Research Center, Forestry and Forest Products Research Institute, 68 Nagaikyutaroh, Momoyama, Fushimi, Kyoto, Kyoto, 612-0855 Japan; 2 Department of Entomology, National Chung Hsing University, 145 Xingda Rd., Taichung, 40227 Taiwan; 3 Institute of Molecular Biology, Academia Sinica, Taipei, Taiwan

**Keywords:** Microbial ecology, Entomology

## Abstract

Fungus-growing termites forage dead plant materials from the field to cultivate symbiotic *Termitomyces* fungi in the nest. Termite foraging behavior and the entry of symbiotic arthropod inquilines may transfer nematodes into a nest and adversely affect fungus production. To test whether nematodes were transferred to fungus gardens by termites and inquilines, we examined the occurrence of nematodes in fungus gardens, five termite castes, and nine species of inquilines of a fungus-growing termite, *Odontotermes formosanus*. Our results revealed that nematodes were commonly carried by foraging termites and beetle inquilines. Numerous nematodes were found under the beetle elytra. No nematodes were found on termite larvae, eggs, and wingless inquilines. In addition, nematodes rarely occurred in the fungus garden. By observing the response of nematodes to three species of *Termitomyces* spp. and the fungus gardens, we confirmed that the fungus and fungus gardens are not actually toxic to nematodes. We suggest that nematodes were suppressed through grooming behavior and gut antimicrobial activity in termites, rather than through the antimicrobial activity of the fungus.

## Introduction

### Insect agricultural systems

Agricultural systems are not only reported in humans but also in insects^1,2^. For example, attine ants (Hymenoptera: Formicidae) cultivate a wide range of fungal species^3,4^. Fungus-growing termites (Blattodea: Termitidae) cultivate fungal species of the genus *Termitomyces* (Agaricales: Lyophyllaceae)^5,6^. In addition, ambrosia beetles are wood-boring weevils that cultivate symbiont fungi in their tunnels as sources of nutrition, and such farming behavior has independently evolved more than eleven times in weevils^7,8^. Symbiosis between fungi and farming insects is obligate, and farming insects have evolved specific agricultural behaviors that are comparable to those of humans, such as seeding, cultivation, and harvesting of fungal products for food^1^. For instance, workers of fungus-growing termites forage outside the nest and bring plant materials into the nest. Incoming materials are chewed up by young workers, ingested, passed rapidly through their gut with little digestion, and then deposited as feces on the top rim of the fungus garden. At the same time, vegetative fungus spores are ingested from conidiophores (=vegetative fruiting bodies) produced on slightly older fungus garden, to inoculate the newly-deposited materials^2,9,10^. The termites then harvest aged substrates and fungal nodules growing on the surfaces of fungus gardens for food^11^. The symbiont fungi are a major source of nutrition for farming insects since the fungi hold nutrients critical for their growth, including essential vitamins, amino acids, and sterols^2,12–14^.

### Nematodes as potential pests in agricultural systems of termites

Nematodes are found in various terrestrial environments^15^ and are widely regarded as agricultural pests. For example, plant-parasitic nematodes cause severe losses to crop production and are vectors of plant pathogens^16,17^. Nematodes are also considered as pests in both human^18,19^ and insect^20^ fungicultural systems. Termites have commonly been reported to carry nematodes, mostly microbe-feeders in the phoretic stage^21–29^. In addition, nematode species associated with termites differ from those randomly sampled from soil or epiphytic nematodes, which implies that termite-associated nematodes are transferred by termites and potentially other soil invertebrates as well, and inhabit the carriers’ habitats and/or nests^30,31^. Invasion of termite nests by nematodes is possible and may lead to production losses through the consumption of fungi and fungus gardens. Fungus gardens of termites are rich in carbohydrates, proteins, and lipids^10,32^. Fungus gardens are not only media for growing *Termitomyces*, the symbiont fungus, but also media for growing other fungal species, such as *Xylaria* fungi (Xylariales: Xylariaceae)^33^, and are potential media for the growth of bacteria and nematodes.

### Transmission and management of pests in insect agricultural systems

Microbes that are pathogenic to fungi are potential pests in agricultural systems and are potentially transferred to fungus gardens when (1) insect foragers carry materials with microbes from extranidal environments and contaminate the cultivating substrates and fungus; (2) numerous “guests” (inquilines), such as myrmecophiles, associated with ants, and termitophiles, associated with termites^34–37^ enter nests of social insects through chemical mimicry or chemical insignificance^38,39^, and may carry pathogenic microbes into the agricultural system; and (3) pathogens are vertically transmitted through colonizers, such as the alates of ants and termites, carrying pathogens from parental colonies^1^ to newly founded colonies.

Pest management strategies have also been reported in agricultural systems of insects. For example, in fungus gardens of leaf-cutter ants, the parasitic microfungus *Escovopsis* (Hypocreales: Hypocreaceae) are suppressed by antimicrobial chemicals produced by actinomycete bacteria, a symbiont of leaf-cutter ants^40–42^. Um, *et al*.^43^ also observed that a strain of *Bacillus* in fungus gardens of termites suppressed non-*Termitomyces* microbes. Allogrooming behavior among nest mates, which has been reported in termites and ants, may remove pathogens from body surfaces^2,44,45^. In termites, partitioning of foraging and nest-caring tasks among individuals has been suggested to inhibit the transfer of pathogens in colony^46^. Fungus-growing termites were also hypothesized to suppress non-*Termitomyces* microbes by passing fungus garden materials through their guts, which generally have high levels of antimicrobial activity^47^.

### Purpose of this study

We aimed to understand the transmission and management of nematodes in an agricultural system of a fungus-growing termite, *Odontotermes formosanus* (Shiraki). We investigated three transmission pathways of nematodes in the agricultural system: (1) vertical transmission via alate, (2) horizontal transmission via foragers, and (3) horizontal transmission via inquilines. In addition, we assessed the potential of fungus gardens as media for nematodes. We also examined factors that may suppress nematode populations in termite nests.

## Materials and Methods

### Hosts of nematodes

**Figure 1:**
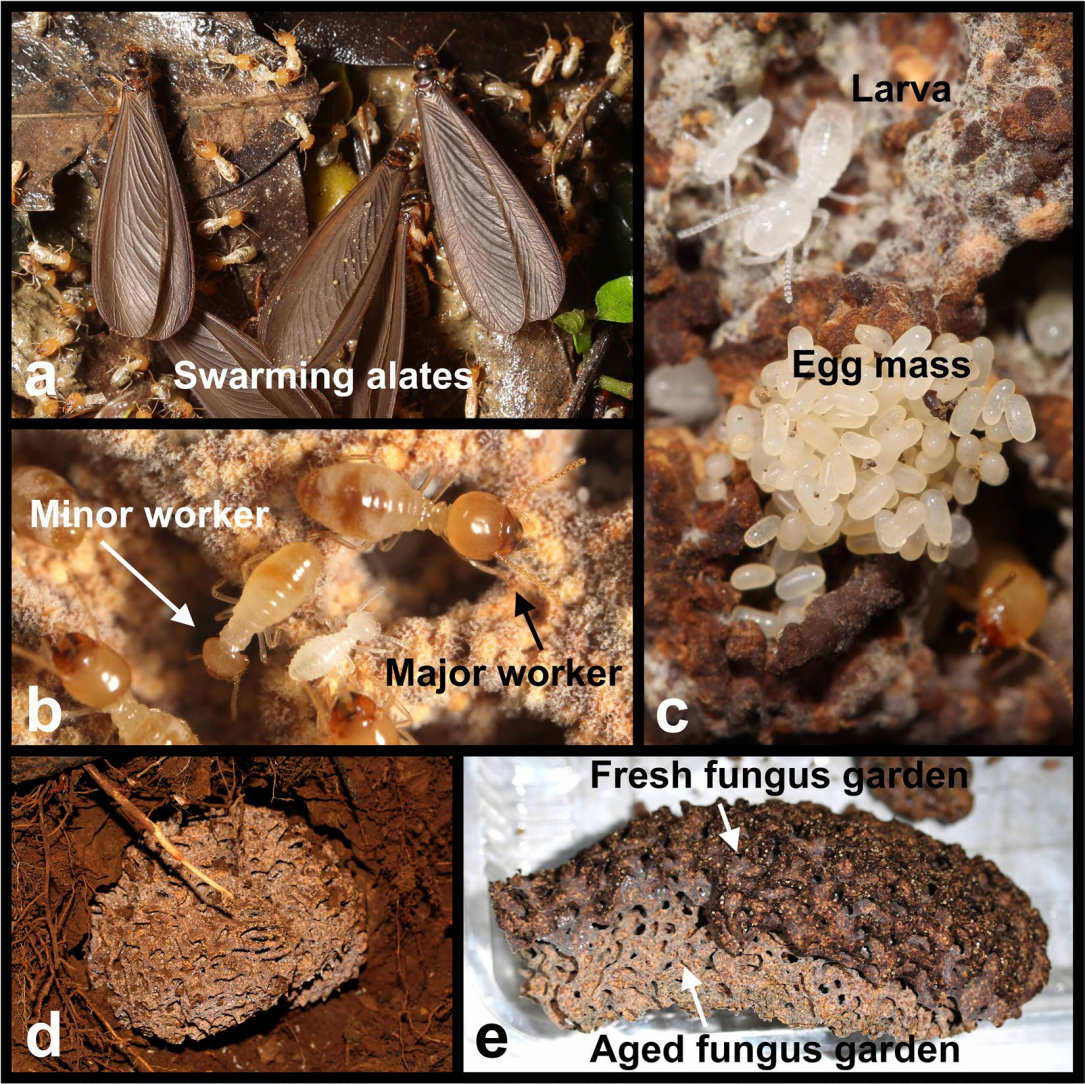
Major components of *Odontotermes formosanus* agriculture. (**a**) Swarming alates; (**b**) minor and major workers; (**c**) termite larvae and egg mass; (**d**) a fungus comb *in situ* in the soil, part of a large nest of *O*. *formosanus*; (**e**) an *O*. *formosanus* fungus garden (note that the dark upper layer is comprised of fresh plant material and the pale lower layer is aged and decomposed material).

**Figure 2:**
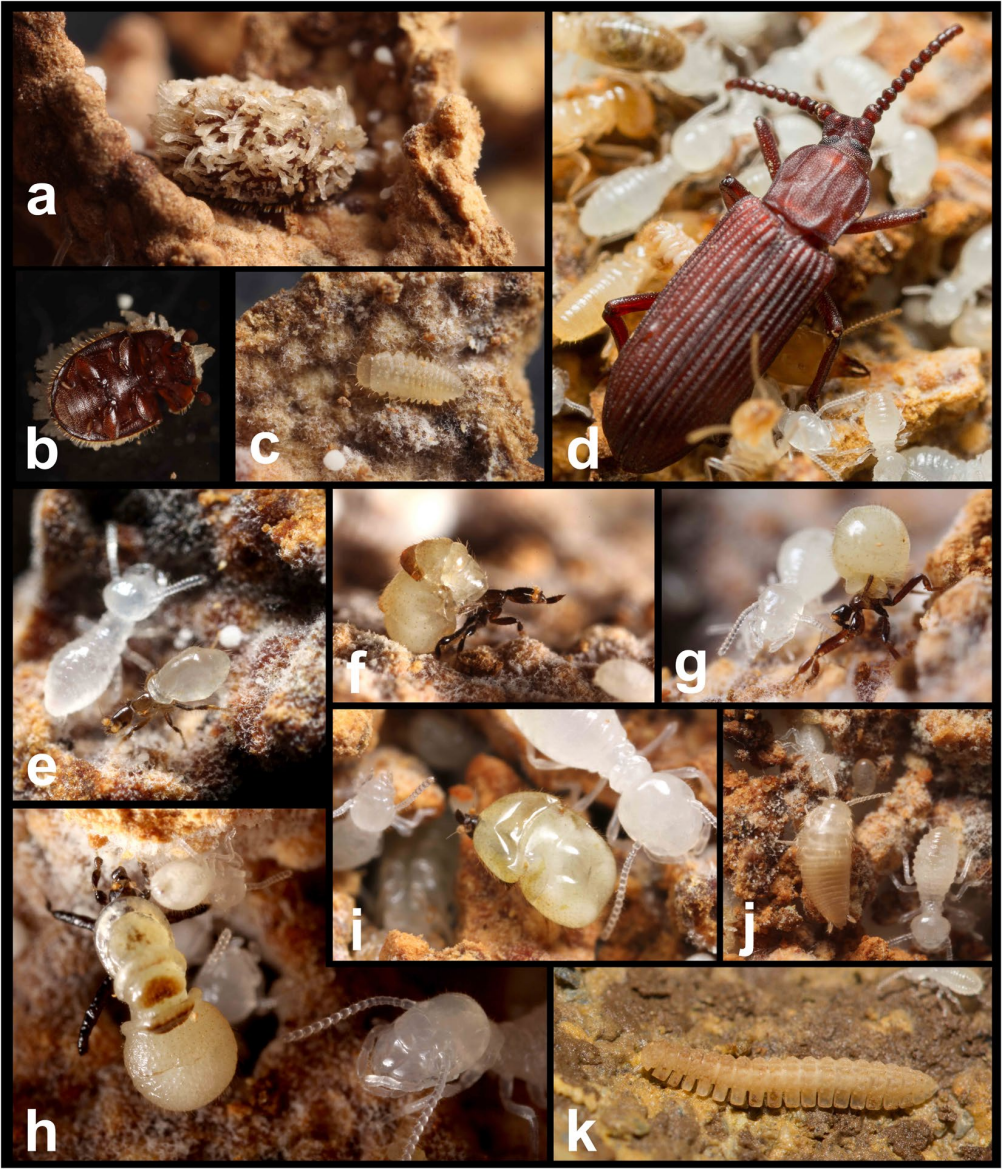
Inquilines of *Odontotermes formosanus*. (**a**) *Cycloxenus* sp. (Coleoptera: Cerylonidae), adult in dorsal view; (**b**) *Cycloxenus* sp., adult in ventral view; (**c**) larva of *Cycloxenus* sp.; (**d**) *Ziaelas formosanus* (Coleoptera: Tenebrionidae); (**e**) *Clitelloxenia audreyae* (Diptera: Phoridae); (**f**) *Clitelloxenia formosana* (Diptera: Phoridae); (**g**) *Selenophora shimaidai* (Diptera: Phoridae); (**h**) *Pseudotermitoxenia nitobei* (Diptera: Phoridae); (**i**) *Horologiphora sinensis* (Diptera: Phoridae); (**j**) *Platystylea* sp. (Thysanura: Nicoletiidae); (**k**) millipede (Diplopoda: Polydesmida: Pyrgodesmidae).

Five nests of *Odontotermes formosanus* from different localities in Taiwan were located by searching for fruiting bodies (mushrooms) of *Termitomyces* on the ground and were excavated to investigate their potential as hosts of nematodes in nests, including fungus gardens, termites, and inquilines (Figs 1 and 2; Supplementary Information, Table S1); 3–7 fungus gardens were collected from each nest. Since the compositions of microbes may vary between the upper and the lower parts of fungus gardens^47^, 6 g from the upper and lower parts were collected separately (Supplementary Information, Table S2) to investigate the presence of nematodes. The upper parts of fungus gardens are dark-colored fresh substrates, comprising partially-digested plant material. By contrast, the lower parts are whitish aged substrates, comprising dense fungal hyphae and highly decomposed plant materials (Fig. 1e)^48^. Between the dark-colored fresh substrates and the whitish old substrates is the active zone in which the *Termitomyces* conidiophores are mainly produced. The upper and lower parts were sampled separately by dividing the active zone at the middle, and therefore the relatively fresh and aged active zone will be sampled and included in the upper and lower parts, respectively.

Termites and inquilines in each nest were sought and collected exhaustively. To investigate whether the foraging termites carry nematodes, foraging termites on feeding sites (logs, leaves, tree bark) found within 20 m of nests were collected. Feeding sites of *O*. *formosanus* were distinguished by the presence of soil-sheeting, which is specifically built by foragers of *O*. *formosanus*. Inquilines in *O*. *formosanus* nests were identified based on the morphological descriptions of termitophilous insects in Taiwan^49^ (Fig. 2). To examine whether inquilines carry nematodes in the dispersal stage, dispersing adults of a termitophilous beetle, *Ziaelas formosanus* Hozawa (Coleoptera: Tenebrionidae) (Fig. 2d) were collected using a light trap at Xiaping Tropical Botanical Garden, the Experimental Forest, National Taiwan University, Nantou, Taiwan (23.77°N, 120.67°E). *Ziaelas formosanus* is commonly observed in fungus gardens of *O*. *formosanus*. Dispersing termite alates (Fig. 1a) were collected from four localities (Supplementary Information, Table S3) to examine whether nematodes were transferred from parental colonies. Voucher specimens of nematodes, termites, and inquilines were deposited at National Chung Hsing University (NCHU).

### Isolation of nematodes

Fungus gardens, eggs, larvae, major workers, minor workers, and alates of termites were deposited on 2% agar plates (ø = 90 mm)^29^, squashed with tweezers, and observed using a Leica® M205 C stereomicroscope or Leica® DM750 microscope (Leica, Wetzlar, Germany) to inspect the presence of nematodes. To clearly observe nematodes on agar plates, termite eggs were evenly spread on agar plates. Head capsules and digestive systems of termite larvae, workers, and alates were further dissected. To avoid high densities of body parts interfering with the observation of nematodes, the number of larvae and major and minor workers on each agar plate was limited to 50 individuals, and that of termite alates was limited to 40 individuals (20 males and 20 females). Nematodes of inquilines were examined using the same methodology applied to termites. The number of inquilines on each agar plate was approximately 30 individuals. All inquilines were examined on a single agar plate if the nest contained less than 30 individuals. The fungus gardens, eggs, dissected bodies of termites, and inquilines were kept on agar plates for one month at room temperature to allow nematodes to propagate for inspection. The percentage of nematodes present in fungus gardens, termite castes, and inquilines was calculated by dividing the number of plates with nematodes with the total number of plates examined and multiplying by 100.

### Morphotyping and molecular characterization of nematodes

When nematode propagation was confirmed on agar plates, the nematodes were removed manually, their morphologies assessed, and were transferred to nematode digestion buffer^50,51^ for molecular identification. However, if the number of individuals was sufficient to establish cultures, they were transferred to artificial media (i.e., nematode growth medium inoculated with OP50 *Escherichia coli* strain for bacteria feeders or 2% malt extract agar inoculated with *Botrytis cinerea* Pers. for fungal feeders). Cultured nematodes were kept as laboratory strains and the detailed taxonomic studies are presented elsewhere.

Since all nematodes collected during dissection were dauer (dispersal) juveniles that do not have genus/species-specific morphological characters, they were transferred to nematode digestion buffer and molecularly characterized based on ribosomal RNA sequences, that is, near full-length 18S (SSU) and/or D2-D3 expansion segments of 28S (D2-D3 LSU) regions. Molecular sequences were determined through PCR-based direct sequencing according to Kanzaki and Futai^52^ and Ye, *et al*.^53^. Generated sequences were compared with those deposited in the GenBank database (http://www.genome.jp/dbget-bin/www_bfind?genbank-today) using the BLAST homology search program (http://blast.ddbj.nig.ac.jp/blastn?lang=en).

### Fungus toxicity bioassay

To confirm whether the fungal symbiont *Termitomyces* spp. suppressed nematode populations, three *Termitomyces* spp. (Supplementary Information, Table S4) were isolated and cultured on Potato Dextrose Agar (PDA) to examine their nematocidal activity against four nematode genera isolated from termites, fungus gardens, or inquilines. *Termitomyces* spp. strains and their identification based on the internal transcribed spacer (ITS) region were provided by Mycology Laboratory of NCHU (Supplementary Information). Fungus cultures were inoculated with five individuals of one species from each nematode genus. To test whether nematocidal activity was present on fresh and aged substrates of fungus gardens, three fungus gardens were collected from NCHU and separated into fresh and aged parts. Six grams of fresh or aged substrates were vortexed for homogenization and each of the 0.3-g substrates placed on different 2% agar plates (ø = 50 mm). Each plate was inoculated with 10 individuals of a single nematode species. Locomotion and pharyngeal pumping in the nematodes were examined at 1, 24, or 48 hours after exposure to fungal hyphae to observe if the fungal culture would exhibit any toxicity to the nematodes.

## Results

### Nematodes isolated

Seven genotypes of nematodes were recognized from the culture plates, namely, a fungal feeder, *Aphelenchoides* sp. and 6 bacteria feeders, comprising three types of *Acrostichus* sp. (Type A, B, and C), 2 types of *Halicephalobus* spp. (Type A, B), and a *Diplogastrellus* sp. The molecular sequences, BLAST results, and notes on each genotype determined in the present study are presented in Supplementary Information.

No nematodes were observed when dissecting 21 fresh and 21 aged substrates of fungus gardens (Table S2). A few *Halicephalobus* sp. (type B) were observed in only one plate 3 weeks after inoculation of the fresh substrate from Xiaping (1 plate detected/21 plates cultured, ~4% occurrence) (Table S2). No nematode was observed in the plates hosting the aged substrates (0/21 plates, 0%) (Table S2). The results indicate that termite fungiculture systems were almost nematode free.

No nematodes were isolated from the 160 swarming alates (80 males and 80 females) of *O*. *formosanus* from the four localities that were examined (Tables S3 and S5) (0/4 plates, 0%); the results indicated that nematodes were not transferred from parental to incipient colonies. Therefore, within-colony transmission of nematodes via alates was unlikely.

Nematodes were not found when dissecting the termite workers collected in or outside the nests. After culturing on plates, four nematode genotypes were identified from foraging major workers (Supplementary Information, Table S5): an *Aphelenchoides* sp., a *Diplogastrellus* sp., and two genotypes of *Halicephalobus* spp. (type A and B) (3/6 plates, 50%) (Table S5). The major workers collected in the nest hosted three nematode genotypes, namely *Diplogastrellus* sp., *Halicephalobus* spp. (type A and B) (4/7 plates, 57%) (Table S5). Minor workers in nests hosted two nematode genotypes (*Aphelenchoides* sp. and *Diplogastrellus* sp.) (1/5 plates, 25%) (Table S5). No nematodes were detected in termite eggs (0/6 plates, 0%) or larvae (0/13 plates, 0%) (Table S5). The results suggest that nematodes are largely transferred to nests by major workers, which forage and bring plant materials to nests. Therefore, it is likely that horizontal transmission of nematodes via foragers occurred frequently.

**Figure 3:**
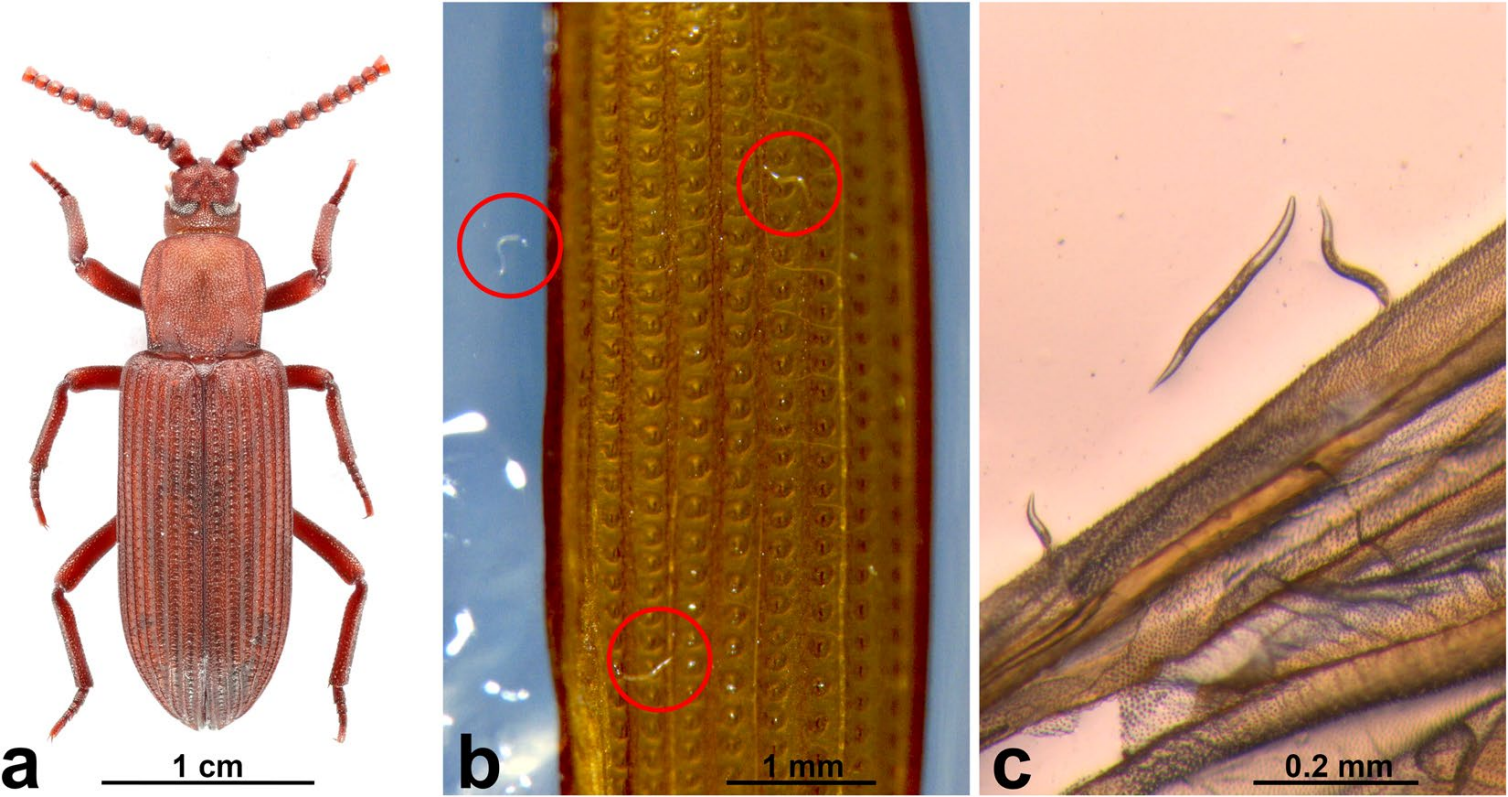
Distribution of nematodes on the body of *Ziaelas formosanus*. (**a**) The habitus of *Ziaelas formosanus*; (**b**) nematodes on the ventral side of the beetle’s elytra; (**c**) nematodes on the beetle’s hindwing.

Nematodes were examined in nine inquiline species previously described by Liang and Li^49^: two coleopteran inquilines, *Cycloxenus* sp. (Cerylonidae) (Fig. 2a–c) and *Ziaelas formosanus* (Tenebrionidae) (Fig. 2d); five dipteran inquilines of the family Phoridae, *Clitelloxenia audreyae* (Fig. 2e), *C*. *formosana* (Fig. 2f), *Selenophora shimaidai* (Fig. 2g), *Pseudotermitoxenia nitobei* (Fig. 2h), and *Horologiphora sinensis* (Fig. 2i); one thysanuran inquiline, *Platystylea* sp. (Thysanura: Nicoletiidae) (Fig. 2j), and a newly recorded species of termitophilous millipede (Diplopoda: Polydesmida) (Fig. 2k) (Table S6). The sample sizes of each of the species or developmental stages of inquilines that were investigated are listed in Table S6. Three nematode genotypes (bacteriophagous *Acrostichus* sp. type A, B, and C) were isolated from adults of *Z*. *formosanus* and *Cycloxenus* sp. (Table S6), and no nematodes were observed in plates hosting larvae of *Cycloxenus* sp. and those of the other inquilines (Table S6). Most nematodes isolated from *Z*. *formosanus* adults were dauer juveniles, inhabiting the section beneath the elytra (Fig. 3). Based on the results, nematodes were transferred by coleopteran inquilines of termites. However, nematodes cultured from coleopteran inquilines and from termites were different.

**Figure 4:**
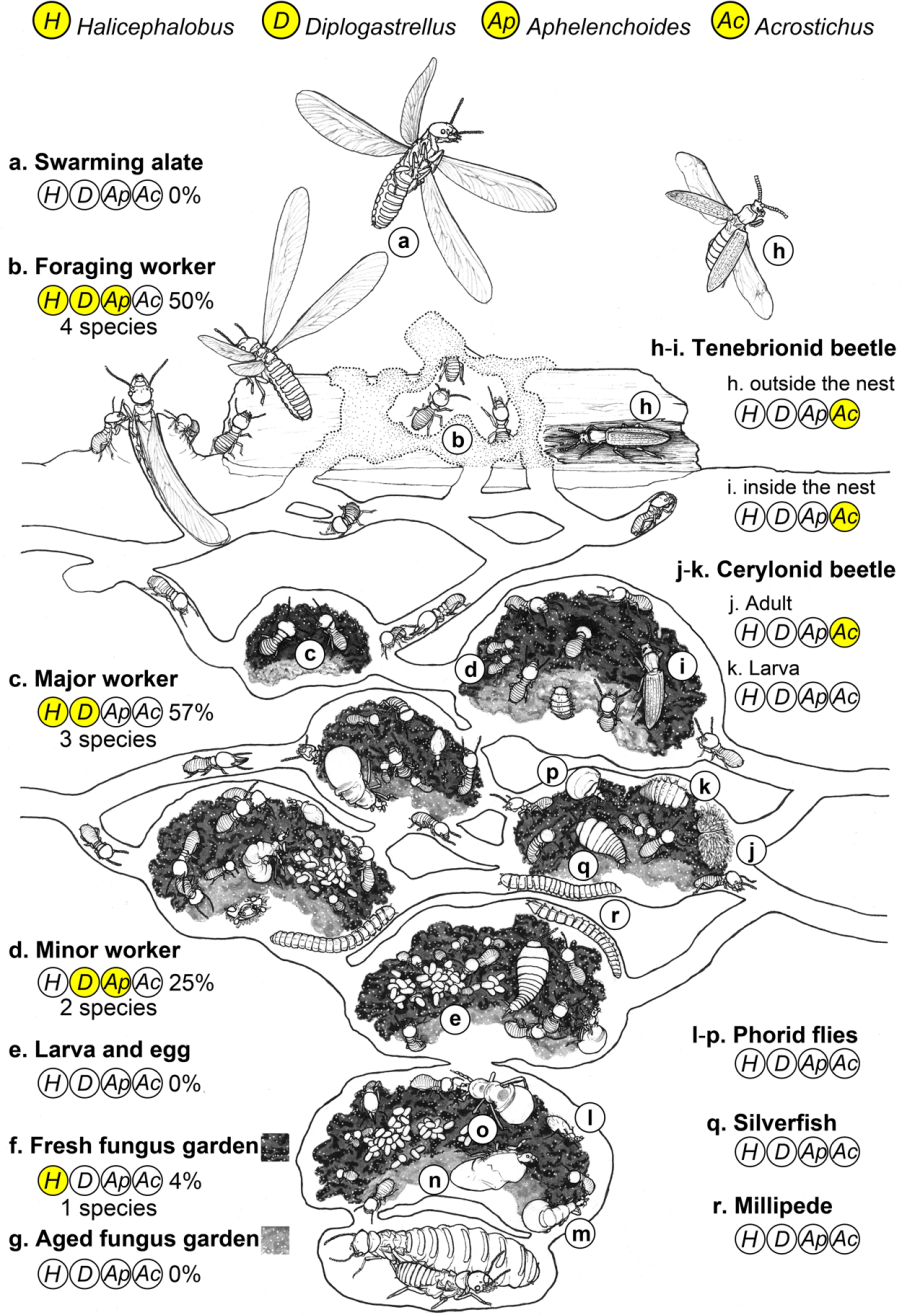
Occurrence of nematodes in *Odontotermes formosanus* termite society and its inquilines. Encircled abbreviations: *H*, nematode genus *Halicephalobus*; *D*, nematode genus *Diplogastrellus*; *Ap*, nematode genus *Aphelenchoides*; *Ac*, nematode genus *Acrostichus*. Yellow circles mean nematodes are present; white circles represent absence of nematodes. Numbers of nematode species and their occurrence rates are denoted for each component of the termite society. Vertical transmission through reproductive castes (4a) to egg or larvae (4e) was not observed. Different nematode species found on termites (4bcd) and inquilines (4hij) indicates that nematodes could enter termite nest through two exclusive horizontal transmission pathways. Extremely low occurrence of nematodes on vulnerable fungus gardens (4fg) indicates termite hygienic behavior plays a critical role in nematode control in the nest.

General information on presence of nematodes on termites, inquilines, and fungus gardens is summarized in Fig. 4. The results revealed that although nematodes were transferred to fungus gardens, the populations were suppressed before or after they entered fungus gardens.

### Fungus toxicity bioassays

Five isolated nematode genotypes, including *Acrostichus* sp., *Aphelenchoides* sp., *Diplogastrellus* sp., and *Halicepholobus* spp., were successfully cultured, (Table S7). No nematodes died or exhibited repellent responses to *Termitomyces* or fungus gardens within 3 hours of inoculation (0/24 plates, 0%). In addition, a fungus-feeding nematode, *Aphelenchoides* sp., was repeatedly observed boring into the fungus garden (Supp. Video 1. https://youtu.be/BIobuqNaw60). The results suggested *Termitomyces* and fungus gardens are not likely to suppress nematodes by themselves.

## Discussion

### Transmission and management of nematodes

The results of the present study revealed that nematodes commonly enter termite nests via two sources: (1) termite foraging workers and (2) coleopteran inquilines. However, being a potential pest, nematodes were not transferred to other castes after entering nests, considering the absence of nematodes on swarming alates, larvae, and eggs. In addition, although nematodes were not repelled by fungus and fungus gardens, they rarely occurred on fungus gardens, which indicated that nematodes were likely suppressed before entering fungus gardens. We suggest that the low quantities of nematodes in agricultural systems of fungus-growing termites were due to integrated management by termites through three strategies: (1) prevention of transmission of nematodes by partitioning of tasks and diets; (2) suppression of nematodes in fungus gardens by passing fresh substrates through their guts; and (3) decontaminating the vectored nematodes through allogrooming behavior.

In fungus-growing termites, major workers are generally foragers while minor workers are mostly nest-keepers. For example, in a fungus-growing termite, *O*. *distans* Holmgren and Holmgren, 94.8% of the individuals collected from feeding sites were major workers, and 95.1% of the individuals collected from queen chambers were minor workers^54^. Similarly, in another fungus-growing termite, *Macrotermes subhyalinus* Rambur, 88.8% of foragers were major workers, and 56.1% of individuals in the nest were minor workers^55^. Such partitioning of tasks predictably decreases the level of interactions between major workers and queens, larvae, or eggs, and may lower the probability of transferring nematodes. In addition to task partitioning, age polyethism on diets was also observed in *M*. *subhyalinus*. Young major workers that molted less than 30 days before stay in the nest, consume the plant material collected by foragers, and construct fungus gardens, while the older major workers that molted more than 30 days before are more likely to forage for plant materials in the field and largely consume aged substrates of fungus gardens in their diets^55^. The diet partitioning in fungus-growing termites separates the constructors of fungus gardens and the foragers, which likely decreases the probability of foragers contaminating fresh substrates.

Thomas^47^ reported that an average of 229.8 and 8.6 fungal isolates were found in a gram of plant material collected and in fresh substrate of fungus gardens constructed by the fungus-growing termite, *M*. *bellicosus* (Smeathman), respectively, which supported the claim that microbial populations were suppressed after passing the gut of major workers. We propose that *O*. *formosanus* controlled the populations of nematodes inhabiting the plant materials collected by integrating the task and diet partitioning, and suppressing the microbial populations in fresh substrates of fungus gardens.

Allogrooming behavior, which cleans microbes growing on body surfaces^56–59^, was reported in a fungus-growing termite, *Macrotermes michaelseni* (Sjöstedt)^60^. Suppression of microbial populations by allogrooming behavior has been confirmed in multiple groups of termites, such as genus *Reticulitermes* (Blattodea: Rhinotermitidae)^61^ and genus *Zootermopsis* (Blattodea: Archotermopsidae)^59^. In the present study, no nematodes were observed on the body surfaces of alates, workers, larvae, eggs, and most of the inquilines. Even in the millipedes, which commonly harbor internal parasites^62–64^ and external phoretic nematodes^65^, no nematodes were isolated. For inquilines, nematodes were only found beneath the elytron of coleopteran inquilines, which is a site that is not likely to be cleaned by allogrooming behavior in termites. In the laboratory, we observed that *O*. *formosanus* performed allogrooming behavior on all inquilines (unpublished data). We propose that allogrooming behavior in the fungus-growing termites not only managed the populations of nematodes carried by termites, but also the populations carried by inquilines.

### Diversity of nematodes of termites and inquilines

Three nematode genera were isolated from *O*. *formosanus*: *Halicephalobus*, *Diplogastrellus*, and *Aphelenchoides*. *Halicephalobus* is one of the most ubiquitous nematode genus, inhabiting various environments^66–69^. The genus *Diplogastrellus* is commonly found in rotten plants^70^ and insects associated with rotten plants^71^, as well as in humid soils that are rich in organic matter^72^. Similarly, the genus *Aphelenchoides* sp. is associated with humid soils that are rich in organic matter. The nematode species composition in *O*. *formosanus* is likely associated to soil environment, similar to in termites foraging or nesting in soil. For example, *Halicephalobus* was isolated from the subterranean termite *Reticulitermes lucifagus* in Corsica^23^, from dry/dampwood termites in both Florida and Taiwan^27,73^, from *Coptotermes formosanus* in Florida^74^, and from many different termite species in Central America^30^. *Aphelenchoides* was isolated from two termite species: *Reticulitermes lucifagus* in Corsica^23^ and *Hodotermopsis sjostedti* in Japan^75^. We suggest that the nematodes isolated from termite workers were from their foraging environments, e.g., soils and/or substrates that the termites foraged.

Only a single nematode genus, *Acrostichus*, was isolated from inquilines, which was not found in termites. The result indicated that nematodes were not transferred between termites and inquilines, and the sources of nematodes in inquilines were different from the sources of nematodes in termites. Nevertheless, the life cycle and ecology of *Acrostichus* spp. is unknown.

### Conclusion

Vertical transmission of nematodes through termite dispersal alates to eggs and larvae was not observed in the fungus-growing termite, *O*. *formosanus*. Nematodes were transferred into termite nests through two horizontal transmission pathways, including via termite foragers and inquilines. Nematode species observed on termite foragers are associated with the soil environment, but inquilines carried specific nematode species, which indicates the two horizontal transmission pathways are exclusive. Laboratory experiments revealed neither fungus garden substrates nor *Termitomyces* fungi were toxic to nematodes, but nematodes were barely present in fungus gardens in the field, which indicates termite hygienic behavior plays a vital role in nematode management in the nests.

## Supplementary information

Aphelenchoides sp. bored into termite fungus garden

Supplementary data of collection and identification of nematodes, fungi, inquilines

## Data Availability

The sequences of nematodes and GenBank accession number of *Termitomyces* spp. are available as Supplementary Information to this paper.
