# Supplementary material for: Nematode-free agricultural system of a fungus-growing termite
Source: Sci Rep. 2019 Jun 20;9:8917. doi: 10.1038/s41598-019-44993-8 (PMC6586928; doi:10.1038/s41598-019-44993-8)
Supplement: Supplementary file 2 — Supplementary data of collection and identification of nematodes, fungi, inquilines [file 41598_2019_44993_MOESM2_ESM.docx]

**Supplementary information**

**Nematode-free agricultural system of a fungus-growing termite**

Natsumi Kanzaki^1#^, Wei-Ren Liang^2#^, Chun-I Chiu^2^, Ching-Ting Yang^3^, Yen-Ping Hsueh^3^, and Hou-Feng Li^2*^

^1^ *Kansai Research Center, Forestry and Forest Products Research Institute, 68 Nagaikyutaroh, Momoyama, Fushimi, Kyoto, Kyoto, 612-0855 Japan.*

^2^ *Department of Entomology, National Chung Hsing University, 145 Xingda Rd., Taichung, 40227, Taiwan.*

^3^ *Institute of Molecular Biology, Academia Sinica, Taipei, Taiwan.*

^#^ Contributed equally.

*Corresponding author: e-mail: houfeng@nchu.edu.tw

**Supplementary information:**

**1. Remarks on generic identification of nematodes**

***Aphelenchoides* sp.**

Based on typological characters, i.e., well-developed stylet with basal swelling, well-developed metacorpus (= median bulb), pharyngo-intestional junction located just posterior to median bulb and relatively simple (= without branched spermatheca) gonadal system, the species was identified as *Aphelenchoides* sp. Based on its sex ratio (no male was found from culture regardless of examination of more than 1000 individual adults) and absence of sperm in female spermatheca, the species is considered to have parthenogenetic reproductive mode.

The SSU of *Aphelenchoides* sp. was close to those of several *Aphelenchoides* spp., namely unidentified *Aphelenchoides* spp. (EU287589, DQ901550) and *A. bicaudatus* (KX345119, AY284643, JN887884, JN887885), which was originally described from soil of paddy field, i.e., relatively rich and wet soil environment ^1^.

***Halicephalobus* spp*.***

Those three D2-D3 genotypes are morphologically identical to each other, and have typical characters of the genus *Halicephalobus*, i.e., small body with simple tube-like stoma, well-developed median and basal bulb and female gonadal system possessing single ovary which has dorsally reflexed distal end of the species As is above *Aphelenchoides* sp., lack of male and lack of sperm in adult female gonad suggest that all three genotypes of *Halicephalobus* are parthenogenetic. Three different D2-D3 genotypes of *Halicephalobus* spp. had identical SSU sequence which was close to *H. gingivalis* cryptic species complex, i.e., *H. gingivalis* (KF765478, AF202156, JX674039), *H.* cf. *gingivalis* (HQ697250, JF706244).

***Diplogastrellus* sp.**

The genus *Diplogastrellus* is suspected to be paraphyletic, and supposed to be separated into two genera, *Diplogastrellus* and *Metadiplogaster* ^2^. Based on molecular sequence similarity, the species found in the present study belongs to *Metdiplogaster* clade.

Morphologically, the species was identified by its stomatal morphology, tube-like stoma with dorsal flap-like tooth and subventral ridges, single female gonad and several male tail characters, e.g., spicule and gubernaculum morphology ^3^. The SSU of *Diplogastrellus* sp. was close to several other *Diplogastrellus* spp., e.g., unidentified *Diplogastrellus* spp. (AB597239, KJ877205) and some other diplogastrid genera, *Pseudodiplogasteroides* and *Diplogasteroides*.

***Acrostichus* spp.**

Molecularly, all three D2-D3 genotypes of undescribed diplogastrid genus had slightly different SSU sequences from each other, i.e., there are three different species, and all were close to *Acrostichus* spp., *A. halicti* (U61759), *A. puri* (AB455817), *A. megaloptae* (AB477077-AB477079), unidentified *Acrostichus* sp. (JX163980) and *A. rhynchophori* (AB455210). However, the stomatal morphology of the isolated nematode is clearly different from *Acrostichus*, i.e., the currently isolated species has very long tube-like stoma and fused pro and metacorpus, which are similar to other diplogastrid genera *Rhabditolaimus* and *Mehdinema* ^2-5^. Further, the males have peloderan (wide) terminal bursa, which has not been reported in *Acrostichus*. Interestingly, despite of highly derived stomatal and bursal structure, several other typological characters, e.g., the arrangement of genital papillae, strong body surface striation and female reproductive system possessing vulval opening forming lip-like horizontal slit and receptaculum seminis are common to *Acrostichu.*

===== Sequences, BLAST results and Notes =====

>*Aphelenchoides* sp._ SSU

ATGTGTAAGTCGAGTTGATATAAAGCGAAACTGCGAACGGCTCATTACAaCAGATATAATCTACTTGTTCTTTCTCTTAAACGGATAACTGCGGTAATTCTGGAGCCAATACGTGCCTAAAGCCCGATRCAGCAATGTGTCGGGTGCACTTATCGGAGCAAAATCAATCTTGATTCAATGCTAACTCTGGATAACCCAGCTTATCGCACGCTTCTAAAGCGGCGAAGTGACATTCGAGTATCTGCCCTATCATCTATCGTTGGTAGTGTATTGGACTACCGAGGACGTTACGGGTAACGGAGAATCAGGGTTCGACTCCGGAGAGGGAGCCTTAGAAACGGCTACCATTGCTAAGGCAAGCAGCAGGCGCGCAACTTATCCACTGTCCACTGGGACGAGATAGTGACGAGAAATAACAAGCTCGTTCTCTTTGAGGTCGAGCATTGGAATGGACCGAATGTAAACGATTCGGTGAGTACCCACGAGAGGGCAAGTCTGGTGCCAGCAGCCGCGGTAATTCCAGCTCTCGTAATGTATAGAATTATTGTTGCGGTTAAAAAGTTCGTAGTTGGATTTCATACCGTTTGTGGGTCATTTCTAACGATTTGTACTCGCGACGGTGTTTGCATACAGTAGGCTTGCCTGCTCTATGCGGATTACTTTGAACGGCTTAAGTGCTCAAGGCGAGTCTTCGGACTGAATACCGGTGCATGGAATAATGGAACAAGGTTTCGATCGAGTTTTATTGGTTTTGTCGGTTGAGACAATGGTTAACAGGAACAATTGGGGGCACTCGTATCCCGGCACGAGAGGTGAAATTCGTGGACTGTCGGGAGACGCCCGAAAGCGAAGGCATTTGCcaAGAGTGTTTTCaTTAATCAAGAACGAAAGTCAGAGGTTCGAAGGCGATTAGATACCGCCCTAGTTCTGACCGTAAACGATGCCAACTATCAATCCGCCAGTAGCTTTCAGTTATTGGCGGGCGGATCGACGGAAACGAAAGTTTTTCGGTTCCGGGGGAAGTATGGTTGCAAAGCTGAAACTTAAAGGAATTGACGGAAGGGCACCACCAGGAGTGGAGCTTGCGGCTTAATTTGACTCAACACGGGAAACCTCACCCGGCCAGTACATCATAAGGATTGACAGATTGAAAGCTCTTTCATGATTTGATGAATGGTGGTGCATGGCCGTTCTTAGTTCGTGGCGTGAGTCGTCTGCTTAATTGCGATAACGAGCGAGACTCCGACTGTTAAATAGCACCGGGATCTCTTCGTTCCGGAGGCTTCTTAACTGGATTCGCGGCTTCAAGCCGCAAGAAATGGAGCAATAACAGGTCTGTGATGCCCTTAGATGTTCGGGGCCGCACGCGTGCTACAATGATGGTAACAGCGTGATTGTCCTCGCCCGACAGGGCTTGGCAAACAGTTGAGATATTATTATGGCTGGAATTGAGTGTTGAAATTTTCTCTCATGAACGAGGAATTCCAAGTAAATGCGAGTCATCAACTTGCATTGATTACGTCCCTGCCCTTTGTACACACCGCCCGTCGCTACCCGGGACTGAGTTGTTTCGAGAAATCTACGGACCAGTCAGTTGGAGCTTGCTTCTGCCGGCAGGAAAGTAAATTAATCGCAATGGCTTGAACCGGGT

>*Halicephalobus* sp. (Type A)_D2-D3 LSU

AAAAGTACTCTGAAGAGAGAGTTAAAAGAACGTGAAAACGATCAAGTGGAAGCAAACAGG

TCTATTTGAAGGTATGTAGTTTTCTATATGCCTGCTAATGAGTATATGGGGTTTCTATTA

TTCTGTTTTAAGAGGACTCTTTTAGAGAACCCTTGTGATGGTTGAACGTTATTCTGATAT

ACGAATATTAGCATTTATGATGGTTACCTGTTTGACCCGTCTTGAAACACGGACCAAGAA

GTTTATTGTATACGCGAGTCATTAAGTGTTAAAACTTATTGGCACAATGAAAGTAAAGGT

TCACTTGTTGAGCTGATGTATGACGGATTGTTTACGGACAGTTCAGCAGTACAGTCCTAT

TCTTATGATTTATCATGGAGTGGAGATAGAGCGTATGCAATGAGACCCGAAAGATGGTGA

TCTATGCTTGAGCAGGATGAAGCCGGAGGAAACTCTGGTGAAAGTCCGTAACGGTTCTGA

CGTGCAAATCGATCGTCTGACTTGAGTATAGGGGCGAAAGACTAATCGAACCATC

>*Halicephalobus* sp. (Type B) _D2-D3 LSU

AAAAGTACTCTGAAGAGAGAGTTAAAAGAACGTGAAAACGATCAAGTGGAAGCAAACAGG

TCTATTTGAAGGTATGTAGTTTTCTATATGCCTGCTAATGAGTATATGGGGTTTCTATTA

TTCTGTTTTAAGAGGACTCTTTTAGAGAACCCTTGTGATGGTTGAACGTTATTCTCATAT

ACGAATATTAGCATTTATGATGGTTACCTGTTTGACCCGTCTTGAAACACGGACCAAGAA

GTTTATTGTATACGCGAGTCATTAAGTGTTAAAACTTATTGGCACAATGAAAGTAAAGGT

TCACTTGTTGAGCTGATGTATGACGGATTGTTTACGGACAGTTCAGCAGTACAGTCCTAT

TCTTATGATTTATCATGGAGTGGAGATAGAGCGTATGCAATGAGACCCGAAAGATGGTGA

TCTATGCTTGAGCAGGATGAAGCCGGAGGAAACTCTGGTGAAAGTCCGTAACGGTTCTGA

CGTGCAAATCGATCGTCTGACTTGAGTATAGGGGCGAAAGACTAATCGAACCATC

>*Diplogastrellus* sp. _D2-D3 LSU

CAAAGAACTTTGAAGAGAGAGTTCAAGAGGACGTGAAACCACAAGGGGCGAAACGGATAG

AGTTGACGAGACTGGCGGTGATCAGTGCTGTCGAGGGCTCCGCTGTGCGATCGGCTCCTG

ATGAGGAGTCGCGTGCGGTTTGGGTGAGAACTTGGCGGCGCGCATTCACTGTCGGTATAC

GCTGAGACGTTCGGCTGTCCCTTCTGAACTGTGCGATGTGGCCCACTCTCGGGTGGTCAC

CTGTCGTGCAGGGAGGTTGTTGTGATGGCTGAGCGGCTGGATCGGGCCTCGCCTTTGCGC

GGAGCTTGCGGACGCTCGTCTGCGGCGTTGTTGGTGGCTTGCCATCTGCGACGTTTGTGG

GCTTGTGTACGTGAGTGCTGCTATCAGGCTTGCTCTCGGTGTGAAAGTCGACCACCTATC

CGACCCGTCTTGAAACACGGACCAAGAAGTCTAGTATGTGCGCGAGTCAATGGGTGCAAA

ACCTACTGGCGAAATGAAAGTAAAGGCTGGCTCGACTGGCTGATATGTGATCCGTGGCTA

CGGCTGCGGCGCAACATAGCCCCATGCCGACTGCTTGCAGTGGTGTGGAGGTAGAGCGTA

CATACTGGGACCCGAAAGATGGTGAACTATGCCTGAGCAGGATGAAGCCAGAGGAAACTC

TGGTGGAGGTCCGAAGCGGTTCTGACGTGCAAATCGATCGTCTGACTTGGGTATAGGGGC

GAAAGACTAATCGAACCATC

>*Acrostichus* sp. (Type A) _D2-D3 LSU

AAAAGAACTTTGAAGAGAGAGTTCAAGAGGACGTGAAACCACTTGGATCGAAGCGGATAG

AGTTGACGAAACTGGCGGCATTCAGGTCGTGCGTTGTCGTGATGTGGCGGGTGCCTCTGC

TATGGCGCCGTAAGTCCGCTAATCGGTGGCGTTTCGTACTGCACTTGCTGCCGGTATACG

CTGAGGCGGTCGATTGCCCCTTCCGAAGCGTGCTGTGTTGCCCACCGCGTGTGGTGTCTT

GCGGCGCGTCGAGGTTGATGTGGCGATTGGCTGTTAGCGCGTTCCGTGCCTCTGCGCTTT

GCGTCGTGGCCGGTGCTGTGCGTCGTGTCGGAGGCTTGCCTCTTGGTGCTTCGTGCGGGG

CTGGTGCGGCGCGTTGCGCTTGGAACGTTCTCGGTGTCGAAAAGTCGACCACCTATCCGA

CCCGTCTTGAAACACGGACCAAGAAGTCTAGTATGTGCGCGAGTCATTGAGTGGAAAACT

CATATGGCGTAATGAAAGTAAAGGTTGGCGTTCGCTGACTGATATGTGATCCGTGGGCTT

GCCCGCGGCGCAACATAGCCCCATGCCGATTGCTTGCAATGGTGTGGAGGTAGAGCGTAC

ATACTGGGACCCGAAAGATGGTGAACTATGCTCGAGCAGGACGAAGTCAGAGGAAACTCT

GATGGAAGTCCGTAGCGGTTCTGACGTGCAAATCGATCGTCGGACTTGAGTATAGGGGCG

AAAGACTAATCGAACCATC

>*Acrostichus* sp. (Type B) _D2-D3 LSU

AAAAGAACTTTGAAGAGAGAGTTCAAGAGGACGTGAAACCACTTGGATCGAAGCGGATAG

AGTTGACGAAACTGGCGGCATTCAGGTCGTGCGTTGTCGTGATGTGGCGGGTGCCTCTGC

TATGGCGCCGTAAGTCCGCTAATCGGTGGCGTTTCGTACTGCACTTGCTGCCGGTATACG

CTGAGGCGGTCGATTGCCCCTTCCGAAGCGTGCTGTGTTGCCCACCGCGTGTGGTGTCTT

GCGGCGCGTCGAGGTTGATGTGGCGATTGGCTGTTAGCGCGTTCCGTGCCTCTGCGCTTT

GTGTCGTGGCCGGTGCTGTGCGTCGTGTCGGAGGCTTGCCTCTTGGTGCTTCGTGCGGTG

CTGGTGCGGCGCGTTGCGCTTGGAACGTTCTCGGTGTCGAAAAGTCGACCACCTATCCGA

CCCGTCTTGAAACACGGACCAAGAAGTCTAGTATGTGCGCGAGTCATTGAGTGGAAAACT

CATATGGCGTAATGAAAGTAAAGGTTGGCGTTCGCTGACTGATATGTGATCCGTGGGCTT

GCCCGCGGCGCAACATAGCCCCATGCCGATTGCTTGCAATGGTGTGGAGGTAGAGCGTAC

ATACTGGGACCCGAAAGATGGTGAACTATGCTCGAGCAGGACGAAGTCAGAGGAAACTCT

GATGGAAGTCCGTAGCGGTTCTGACGTGCAAATCGATCGTCGGACTTGAGTATAGGGGCG

AAAGACTAATCGAACCATC

>>*Acrostichus* sp. (Type C) _D2-D3 LSU

AAAAGAACTTTGAAGAGAGAGTTCAAGAGGACGTGAAACCACTTGGATCGAAGCGGATAG

AGTTGACGAAACTGGCGGCATTCAGGTCGTGCGTCGTCGTGATGTGGCGGGTGCCTTGTT

TATGGCGCCATAAGACCGCTAATCGGTGGCGTTTCGTACTGCACTTGCTGTCGGTATACG

CTGAGGCGGTCGATTACCCCTTCCGAAGCGTGCTGTGTTGCCCACCGCGTGTGGAGTCTT

GCGGCGCGTCGAGGTTGATGTGGAGATTGACTGTCAGCGCGTTCCGTGCCTCTGCGCTTT

GCGTCGTGGCCAGTGTTGTGCGTTGTGTCGGAGGCTTGCCTCTTGATGCTTCGTGCGATG

CTGGTGCGGTGCGTTGCGCTTGGAACGTTCTCGGTGTCGAAAAGTCGACCACCTATCCGA

CCCGTCTTGAAACACGGACCAAGAAGTCTAGTATGTGCGCGAGTCATTGAGTGGAAAACT

CATATGGCGTAATGAAAGTAAAGGTTGGCGTTCGCTGACTGATATGTGATCCGTGGGCTT

GCCCGCGGCGCAACATAGCCCCATGCCGATTGCTTGCAATGGTGTGGAGGTAGAGCGTAC

ATACTGGGACCCGAAAGATGGTGAACTATGCTCGAGCAGGACGAAGTCAGAGGAAACTCT

GATGGAAGTCCGTAGCGGTTCTGACGTGCAAATCGATCGTCGGACTTGAGTATAGGGGCG

AAAGACTAATCGAACCATC

===== Close sequences (BLAST search) =====

*Halicephalobus* type A:

MG051262 Halicephalobus sp. AA3 93% (isolated from Coptotermes in USA: Jeremiah's paper)

JX674038 Halicephalobus gingivalis 93%

AB289345 Halicephalobus gingivalis 93%

JQ838156 Halicephalobus cf. gingivalis 92%

MG051261 Halicephalobus sp. AA4 92% (isolated from Coptotermes in USA: Jeremiah's paper)

MG051246 Halicephalobus sp. KW-1A-1 92% (isolated from Coptotermes in USA: Jeremiah's paper)

JX194163 Halicephalobus gingivalis 92%

AY294179 Halicephalobus gingivalis 92%

AB288935 Halicephalobus gingivalis 92%

AY294180 Halicephalobus gingivalis 92%

*Halicephalobus* type B:

MG051262 Halicephalobus sp. AA3 93% (isolated from Coptotermes in USA: Jeremiah's paper)

JX674038 Halicephalobus gingivalis 92%

JQ838156 Halicephalobus cf. gingivalis 92%

MG051261 Halicephalobus sp. AA4 92% (isolated from Coptotermes in USA: Jeremiah's paper)

MG051246 Halicephalobus sp. KW-1A-1 92% (isolated from Coptotermes in USA: Jeremiah's paper)

JX194163 Halicephalobus gingivalis 92%

AB289345 Halicephalobus gingivalis 93%

AY294180 Halicephalobus gingivalis 92%

AY294177 Halicephalobus gingivalis 92%

AY294181 Halicephalobus gingivalis 92%

*Diplogastrellus* sp.:

AB597250 Diplogastrellus sp. NK-2010 98% (isolated from Reticulitermes in Japan: Kanzaki unpubl.)

KJ877248 Diplogastrellus sp. VS-2014 97%

AB597248 Pseudodiplogasteroides cf. compositus 95%

AB597249 Pseudodiplogasteroides sp. NK-2010 94%

LC099975 Diplogasteroides luxuriosae 92%

LC145090 Diplogasteroides nix 91%

LC099974 Diplogasteroides luxuriosae 91%

AB808723 Diplogasteroides andrassyi 91%

LC027673 Diplogasteroides asiaticus 91%

KJ877247 Butlerius sp. VS-2014 90%

*Acrostichus* sp. type A:

AB477075 Acrostichus puri 88%

LC374587 Acrostichus floridensis 88%

AB455818 Acrostichus halicti 88%

EU195983 Acrostichus halicti 87%

JX163971 Acrostichus sp. RS5083 86%

AB477074 Acrostichus megaloptae 88%

HQ130209 Acrostichus halicti 86%

HQ130221 Acrostichus halicti 86%

HQ130204 Acrostichus halicti 86%

HQ130223 Acrostichus halicti 86%

*Acrostichus* sp. type B:

AB477075 Acrostichus puri 88%

LC374587 Acrostichus floridensis 88%

AB455818 Acrostichus halicti 88%

EU195983 Acrostichus halicti 87%

JX163971 Acrostichus sp. RS5083 86%

AB477074 Acrostichus megaloptae 88%

HQ130209 Acrostichus halicti 86%

HQ130221 Acrostichus halicti 86%

HQ130204 Acrostichus halicti 86%

HQ130223 Acrostichus halicti 86%

*Acrostichus* sp. type C:

AB477075 Acrostichus puri 88%

LC374587 Acrostichus floridensis 87%

EU195983 Acrostichus halicti 87%

AB455818 Acrostichus halicti 87%

JX163971 Acrostichus sp. RS5083 86%

AB477074 Acrostichus megaloptae 88%

HQ130194 Acrostichus puri 85%

HQ130209 Acrostichus halicti 85%

HQ130221 Acrostichus halicti 85%

===== Notes =====

The species identification was primary conducted based on the D2-D3 expansion segments of the large subunit of ribosomal RNA gene.

Two sequence types of *Halicephalobus* spp. were close to several unidentified species isolated from *Coptotermes formosanus* in the United States (Florida) and H. gingivalis isolates with 92-93% of identity.

*Diplogastrellus* sp. was very close (98% identity) to its congener isolated from Reticulitermes spertus from Japan and unidentified Diplogastrellus (97% identity). The species is also close to other diplogastrid species but hte identity is not so high (90-95% of identity).

Three types of *Acrostichus* were all close to Acrostichus spp., but the identity is not so high (85-88%), and it could be highly derived species in the genus.

## 2. Fugal DNA extraction, rDNA barcoding polymerase chain reaction (PCR), DNA sequencing and phylogenetic analyses

Fungal isolates grown on PDA 7-10 days were prepared for DNA extraction. The protocol of DNA extraction was carried out following ^6^. PCR amplification was performed by using experimental sample cocktail, consisting of 2-8 ng DNA template, 0.4 ng upstream primer and downstream primer, PCR Master Mix II (5X) (GeneMark Technology Co., Ltd., Taiwan), each of the resulting 25 µL (1X) PCR reactions contains 0.75U of Taq DNA polymerase, reaction buffer, 2 nM MgCl2, 250µM dNTPs and enzyme stabilizer, in 25µL total volume. For the rDNA barcoding, the primer set used to amplify the internal transcribed spacer (ITS) region was carried out by ITS5 and ITS4, yielding a product of about 600 bp. The amplification of protocol was used: initial denaturation 94 ºC for 2 minutes, followed by 35 cycles of 94 ºC for 30 seconds, then 30 seconds at 50 ºC and 30 seconds at 72 ºC and a final extension of 10 minutes at 72 ºC.

The sequencing of the ITS sequences were achieved by using the same primer sets of PCR amplification. Sequencing was performed on an ABIPRISM 377 DNA sequencer at the Biotechnology Center of National Chung Hsing University.

**References**

1 Imamura, S. Nematodes in the paddy field, with notes on their population before and after irrigation. *Journal of the College of Agriculture, Imperial University of Tokyo* **11**, 193-240 (1931).

2 Susoy, V., Ragsdale, E. J., Kanzaki, N. & Sommer, R. J. Rapid diversification associated with a macroevolutionary pulse of developmental plasticity. *Elife* **4**, e05463 (2015).

3 Sudhaus, W. & Fürst von Lieven, A. A phylogenetic classification and catalogue of the Diplogastridae (Secernentea, Nematoda). *J. Nematode Morphol. Syst.* **6**, 43-90 (2003).

4 Susoy, V. & Herrmann, M. Validation of Rhabditolaimus Fuchs, 1914 (Nematoda: Diplogastridae) supported by integrative taxonomic evidence. *Nematology* **14**, 595-604 (2012).

5 Luong, L. T., Platzer, E. G., De Ley, P. & Thomas, W. K. Morphological, molecular, and biological characterization of *Mehdinema alii* (Nematoda: Diplogasterida) from the decorated cricket (*Gryllodes sigillatus*). *J. Parasitol.* **85**, 1053-1064 (1999).

6 Sambrook, J., Russell, D. W., Janssen, K. & Argentine, J. *Molecular cloning: a laboratory manual on the web*. (Cold Spring Harbor Laboratory, 2001).

**Table S1.** Collection information of termite nests

| Location (abbreviation) | County | Collection date | GPS code (elevation) | Code |
| --- | --- | --- | --- | --- |
| National Chung Hsing University campus (NCHU) | Taichung | 11. Aug., 2016 | 24.119434; 120.674209 (59 m) | TW6250 |
| Xiaping Tropical Botanical Garden, the Experimental Forest,  National Taiwan University (Xiaping) | Nantou | 12. Aug., 2016 | 23.773071; 120.673345 (141 m) | TW6251 |
| Huisun Forest Station, National Chung Hsing University, site 1(Huisun-1) | Nantou | 14. Aug., 2016 | 24.091760; 121.032700 (705 m) | TW6252 |
| Huisun Forest Station, National Chung Hsing University, site 2 (Huisun-2) | Nantou | 15. Aug., 2016 | 24.093100; 121.031480 (736 m) | TW6253 |
| Lienhuachih Research Center, Taiwan Forestry Research Institute (Lienhuachih) | Nantou | 24. Sept., 2016 | 23.917550; 120.884560 (703 m) | TW6254 |

**Table S2.** Nematodes isolated from fungus garden

| Nests | Comb ID | Nutritional status | Nematode isolation |
| --- | --- | --- | --- |
| NCHU | 1 | Fresh | - |
|  |  | Aged |  |
|  | 2 | Fresh | - |
|  |  | Aged |  |
|  | 3 | Fresh | - |
|  |  | Aged |  |
|  | 4 | Fresh | - |
|  |  | Aged |  |
|  | 5 | Fresh | - |
|  |  | Aged |  |
| Xiaping | 1 | Fresh | - |
|  |  | Aged |  |
|  | 2 | Fresh | *Halicephalobus* sp. type B |
|  |  | Aged |  |
|  | 3 | Fresh |  |
|  |  | Aged |  |
|  | 4 | Fresh |  |
|  |  | Aged |  |
| Huisun-1 | 1 | Fresh | - |
|  |  | Aged |  |
|  | 2 | Fresh | - |
|  |  | Aged |  |
|  | 3 | Fresh | - |
|  |  | Aged |  |
| Huisun-2 | 1 | Fresh | - |
|  |  | Aged |  |
|  | 2 | Fresh | - |
|  |  | Aged |  |
|  | 3 | Fresh | - |
|  |  | Aged |  |
|  | 4 | Fresh | - |
|  |  | Aged |  |
|  | 5 | Fresh | - |
|  |  | Aged |  |
|  | 6 | Fresh | - |
|  |  | Aged |  |
|  | 7 | Fresh | - |
|  |  | Aged |  |
| Lienhuachih | 1 | Fresh | - |
|  |  | Aged |  |
|  | 2 | Fresh | - |
|  |  | Aged |  |
|  | 3 | Fresh | - |
|  |  | Aged |  |
|  | 4 | Fresh | - |
|  |  | Aged |  |
|  | 5 | Fresh | - |
|  |  | Aged |  |
|  | 6 | Fresh |  |
|  |  | Aged | - |

**Table S3.** Collection information of swarming alates

| Location (abbreviation) | County | Collection date | GPS code (elevation) | Code |
| --- | --- | --- | --- | --- |
| National Chung Hsing University campus (NCHU) | Taichung | 12. Aug., 2016 | 24.119302; 120.672266 (58 m) | TW6255 |
| National Pingtung University of Science and Technology (NPUST) | Pingtung | 25. May., 2017 | 22.645719; 120.606657 (74 m) | TW6256 |
| Guandaoshan | Miaoli | 27. May., 2017 | 24.373665; 120.810863 (832 m) | TW6257 |
| Huisun Forest Station, National Chung Hsing University (Huisun) | Nantou | 02. Jun., 2017 | 24.090658; 121.031408 (688 m) | TW6258 |

**Table S4.** Collection information of *Termtomyces* spp.

| Code | Accession no. | Location | County | Collection date | Species | Source |
| --- | --- | --- | --- | --- | --- | --- |
| TMGG | LC425109 | Endemic Species Research Institute | Nantou | 22. Aug. 2016 | *Termitomyces microcarpus* | fungus garden |
| BFA | LC425110 | National Chung Hsing University campus | Taichung | 5. Apr. 2016 | *Termitomyces intermedius* | fruiting body |
| 1606SPDF-4 | LC425111 | Xiaping Tropical Botanical Garden | Nantou | 11. Aug. 2016 | *Termitomyces* sp. (undescribed species) | fruiting body |

**Table S5.** Nematodes isolated from termites

| Caste | Location | Plate ID | Fungus garden ID | Nematode isolation |
| --- | --- | --- | --- | --- |
| Egg | Xiaping | 1 | 5 | - |
|  | Xiaping | 2 | 5 | - |
|  | Huisun | 3 | 2 | - |
|  | Huisun-1 | 4 | 3 | - |
|  | Huisun-2 | 5 | 6 | - |
|  | Lienhuachih | 6 | 5 | - |
| Larva | NCHU | 1 | 1 | - |
|  | NCHU | 2 | 2 | - |
|  | Xiaping | 3 | 1 | - |
|  | Xiaping | 4 | 5 | - |
|  | Huisun-1 | 5 | 1 | - |
|  | Huisun-1 | 6 | 2 | - |
|  | Huisun-1 | 7 | 3 | - |
|  | Huisun-2 | 8 | 1 | - |
|  | Huisun-2 | 9 | 2 | - |
|  | Huisun-2 | 10 | 4 | - |
|  | Huisun-2 | 11 | 5 | - |
|  | Lienhuachih | 12 | 5 | - |
|  | Lienhuachih | 13 | 7 | - |
| Minor worker | Xiaping | 1 | 6 |  |
|  | Huisun-1 | 2 | 2 | - |
|  | Huisun-1 | 3 | 3 | *Aphelenchoides* sp., *Diplogastrellus* sp. |
|  | Lienhuachih | 4 | 5 | - |
|  | Lienhuachih | 5 | 7 | - |
| Major worker (in the nest) | Xiaping | 1 | 3 | *Halicephalobus* sp. type B |
|  | Xiaping | 2 | 6 | - |
|  | Huisun-2 | 3 | 3 | *Halicephalobus* sp. type A |
|  | Huisun-2 | 4 | 6 | *Halicephalobus* sp. type B |
|  | Huisun-2 | 5 | 6 | *Halicephalobus* sp. type A, *Diplogastrellus* sp. |
|  | Lienhuachih | 6 | 5 | - |
|  | Lienhuachih | 7 | 7 | - |
| Major worker (foraging worker) | NCHU | 1 | - | *Aphelenchoides* sp. |
|  | NCHU | 2 | - | *Halicephalobus* sp. type B |
|  | Xiaping | 3 | - | - |
|  | Xiaping | 4 | - | - |
|  | Huisun-1 | 5 | - | *Halicephalobus* sp. type A and B, *Diplogastrellus* sp. |
|  | Lienhuachih | 6 | - | - |
| Swarming alate | NCHU | - | - | - |
|  | NPUST | - | - | - |
|  | Guandaoshan | - | - | - |
|  | Huisun | - | - | - |

**Table S6. Nematodes isolated from the inquilines**

| Termitophile species | Location | No. individual examined | Nematode isolation | Note |
| --- | --- | --- | --- | --- |
| Beetles (Hexapoda: Coleoptera) |  |  |  |  |
| Family Cerylonidae |  |  |  |  |
| *Cycloxenus* sp. larvae | Xiaping | 20 | - |  |
| *Cycloxenus* sp. adult | Xiaping | 3 | *Acrostichus* sp. type A |  |
|  | Huisun-1 | 3 | *Acrostichus* sp. type B |  |
|  | Lienhuachih | 29 | *Acrostichus* sp. types B and C |  |
| Family Tenebrionidae |  |  |  |  |
| *Ziaelas formosanus* | Xiaping | 2 | *Acrostichus* sp. type A |  |
|  | Huisun-1 | 2 | *Acrostichus* sp. type B |  |
|  | Lienhuachih | 2 | *Acrostichus* sp. type A |  |
|  | Xiaping | 3 | *Acrostichus* sp. | The beetles were collected outside the termite nest by light trap in 2017/03/31 (TW6260) |
| Flies (Hexapoda: Diptera) |  |  |  |  |
| Family Phoridae |  |  |  |  |
| *Clitelloxenia audreyae* | Xiaping | 1 | - |  |
|  | Huisun-1 | 31 | - |  |
|  | Huisun-2 | 22 | - |  |
|  | Lienhuachih | 13 | - |  |
| *Clitelloxenia formosana* | Xiaping | 4 | - |  |
|  | Huisun-1 | 28 | - |  |
|  | Huisun-2 | 41 | - |  |
|  | Lienhuachih | 30 | - |  |
| *Pseudotermitoxenia nitobei* | Xiaping | 5 | - |  |
|  | Huisun-1 | 8 | - |  |
|  | Huisun-2 | 40 | - |  |
|  | Lienhuachih | 30 | - |  |
| *Horologiphora sinensis* | Huisun-1 | 1 | - |  |
| *Selenophora shimaidai* | Lienhuachih | 31 | - |  |
| Silverfish (Hexapoda: Thysanura) |  |  |  |  |
| Family Nicoletiidae |  |  |  |  |
| *Platystylea* sp. | Huisun-2 | 1 | - |  |
|  | Lienhuachih | 15 | - |  |
| Millipede (Diplopoda: Polydesmida) |  |  |  |  |
| Family Pyrgodesmidae |  |  |  |  |
| Unidentified sp. | Xiaping | 7 | - |  |
|  | Huisun-2 | 9 | - |  |

**Table S7. Nematocidal activity of fungus garden**

| Cultured nematode species | *Termitomyces* spp.* | Fresh fungus garden | Aged fungus garden |
| --- | --- | --- | --- |
| *Acrostichus* sp. (Twof 13) | - | - | - |
| *Aphelenchoides* sp. (Twof 11) | - | -/+ | -/+ |
| *Diplogastrellus* sp. (Twof 12) | - | - | - |
| *Diplogastrellus* sp. (Twof 4-2) | - | - | - |
| *Halicepholobus* sp. (Twof 4: type A) | - | - | - |
| *Halicepholobus* sp. (Twof 5: type A) | - | - | - |
| *Halicepholobus* sp. (Twof 7: type B) | - | - | - |
| *Halicepholobus* sp. (Twof 10: type B) | - | - | - |

*Termitomyces* spp. included 3 strains (see Table S3)

-, no nematocidal activity

+, attractive reaction
